# The Implementation of Infrared Thermography as Complementary Diagnostic Tool in Orthodontic Treatment Plan—Pilot Study

**DOI:** 10.3390/children12121635

**Published:** 2025-12-01

**Authors:** André Brandão de Almeida, André Moreira, Miguel Pais Clemente, Joaquim Mendes, Francisco Salvado e Silva

**Affiliations:** 1 Lisbon Pediatric Dentistry Service (SOL—Serviço Odontopediátrico de Lisboa), Santa Casa da Misericórdia de Lisboa, Largo Trindade Coelho, 1200-470 Lisbon, Portugal; 2 Lisbon Academic Medical Center, Faculty of Medicine, University of Lisbon, 1649-028 Lisbon, Portugal; franciscosalvado@chln.min-saude.pt; 3 Egas Moniz School of Health and Science, Instituto Superior de Ciências da Saúde Egas Moniz, 2829-511 Caparica, Portugal; 4 Faculty of Dental Medicine, University of Porto, 4200-393 Porto, Portugal; andre.luis.sa.moreira@gmail.com; 5 Physiology and Surgery Department, Faculty of Medicine, University of Porto, 4200-319 Porto, Portugal; miguelpaisclemente@hotmail.com; 6 INEGI, Faculty of Engineering, University of Porto, 4200-465 Porto, Portugal; jgabriel@fe.up.pt

**Keywords:** adolescent, cephalometry, infrared thermography, Trevisi, orbicular oris

## Abstract

**Highlights:**

**What are the main findings?**

- Thermographic findings could not be associated with Trevisi cephalometric landmarks in a children-adolescent population.
- Infrared thermography may be a valuable complementary diagnostic tool in orthodontics.

**What is the implication of the main finding?**

- Masticatory muscular thermal asymmetries of the masticatory muscles could not be related with cephalometric landmarks.
- Orbicular oris muscle thermic behavior may be important in the diagnosis of lip incompetence, parafunctional habits and/or hypotonia.

**Abstract:**

Introduction: Infrared thermography (IRT) is a non-invasive, non-ionizing imaging modality capable of rapidly capturing surface temperature variation. In dentistry, particularly orthodontics and TMD evaluation, IRT may serve as a valuable complementary tool to be added in conventional diagnostic protocols. Objective: Correlate possible relationships between thermographic findings of orofacial structures and cephalometric landmarks. Methods: An infrared imaging camera, FLIR^®^ i7, was used to record the regions of interest, correspondent to the temporal, masseter and orbicular oris muscles, in adolescents (*n* = 22). Bilateral temperature differences were considered as thermal asymmetries with a conventional threshold of 0.3 °C to distinguish an eventual hyperactivity or hyperfunctions of detrimental structures. The Trevisi cephalometric parameters that were taken into consideration for the study were SNA, SNB, ANB, OccltoSn, Wits relation to base and Molar/canine classes. Results: Most of the participants showed a normal temperature difference ΔΤ for the upper and lower orbicular oris muscle, right vs. left, 96% and 92%, respectively. The other ROIs presented a mixed pattern of thermal asymmetries; however, no statistically significant differences were found when crossed with the cephalometric landmarks. Conclusions: Asymmetrical patterns of infrared thermography can aid on the diagnosis and treatment plan of an orthodontic appointment, since the actual stability of pos-orthodontic treatment is highly dependent on the muscular activity of the tongue and lips, in particular when the patient has atypical swallowing. Our findings suggest that this technique can be used to quantify anatomical landmarks relevant to craniofacial morphology in specific populations, particularly at ages where muscular functional activity is strongly correlated with dentoskeletal development.

## 1. Introduction

Infrared thermography (IRT) is an imaging modality that can be safely used, as it is non-invasive and uses non-ionizing radiation. Image acquisition is fast (although it requires acclimatization time), the equipment is easily transportable and there are no consumables. These characteristics of thermography generate great potential for use in both engineering and medicine. Naturally, the technique also has limitations; it can only assess surface temperature, and the accuracy of the measurements is strongly affected by the emissivity of the material and environmental conditions. There are numerous applications for infrared thermography in the health sector, ranging from inflammatory arthritis, osteoarthritis, tendonitis, fibromyalgia, complex regional pain syndrome and changes in peripheral vascular microcirculation—such as Raynaud’s phenomenon [1,2], diabetic foot complications [3], temperature screening to assess febrile states, malignant diseases, burns, skin graft areas, dermatological diseases [4] and orofacial pain/temporomandibular disorders [5,6,7,8]. The growing interest in medical thermography is associated with a major evolution in the quality and reliability of existing thermographic cameras, resulting in high-quality, standardized thermal images that can be correlated with clinical findings [9,10]. It should be emphasized that the thermal camera and the thermograms obtained do not allow the clinician to diagnose an existing pathology on their own. However, they do complement both the anamnesis and clinical examination and can thus contribute to a quantitative analysis of certain anatomical structures and regions of interest (ROIs) that can add value to the study of the patient. The data obtained by thermography may be relevant in the field of dentistry for the clinical assessment of malocclusion, particularly in cases of temporomandibular disorders (TMDs). However, this technique can eventually be considered a complementary method of diagnosis in orthodontics in addition to the standardized protocol of collecting study models, performing cephalometry after obtaining a lateral cephalogram and intra- and extraoral photography when studying craniofacial dysmorphia. The standardization and validation of thermography could provide the clinician with a greater number of tools for the initial study of the patient while making it possible to monitor the clinical state throughout the treatment, in terms of asymmetric patterns of the ROIs associated with the corresponding areas of the masticatory muscles, for example. The authors highlight, for instance, the contribution to assessing the effectiveness of speech therapy in patients with atypical swallowing (very typical of Classes II, Div. 1) while eliminating parafunctional tongue habits by monitoring the activity of the lower lip depressor muscle. As is well known, atypical swallowing is usually accompanied by the lower lip overlapping the lower incisors, which is one of the causes of the retroclination of the lower incisal block [11,12].

The main objective of the present investigation was to correlate a possible relationship between thermographic findings of the orofacial structures with cephalometric landmarks. Research question: Can we correlate thermic differences at the masseter, temporal and orbicular oris muscles related to cephalometric findings?

Null hypothesis: The thermographic asymmetries of masseter, temporal and orbicular oris muscles are independent of the cephalometric landmarks.

## 2. Materials and Methods

### 2.1. Participants

Twenty-two participants in the age range of 12–17 years old were enrolled for the study, 10 male and 12 female. All participants were patients from the Lisbon Pediatric Dentistry Service (Serviço Odontopediátrico de Lisboa) of Santa Casa da Misericórdia de Lisboa and Stomatognathic Service (Serviço de Estomatologia) of Hospital Santa Maria. Patients with cleft lip and palate were excluded from the sample.

All participant information was anonymized and randomized. A consent form and explanation of the study was delivered to the adult responsible for the participant.

### 2.2. Data Collection

#### 2.2.1. Infrared Thermography

**Figure 1:**
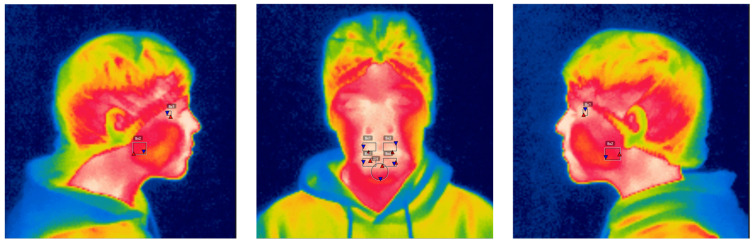
Thermograms, right lateral, frontal and left lateral. On the lateral thermograms the temporal and masseter muscle are being analysed with a square tool. On the frontal thermograph, the upper and lower orbicular oris are being analysed with a square tool, left and right per structure, and the mentalis with a circumferential tool. The geometric forms over the images correspond to the regions of interest (ROIs) analysed.

This project use a Flir^®^ i7 thermographic camera (Wilsonville, OR, USA). The acquisition protocol involved a room without airstreams, direct sunlight, temperature 20–24 °C, humidity 40–60%, without reflective elements, the participant needed 20–30 min to stabilize its body temperature, no make-up, facial creams, hats, earrings, piercings or glasses were allowed. A standard distance of 1 m was used to take the thermograms in a perpendicular direction to the region of interest. A left, frontal and right thermograph was taken of the participant capturing the neck and head region (Figure 1).

#### 2.2.2. Participant Data

**Figure 2:**
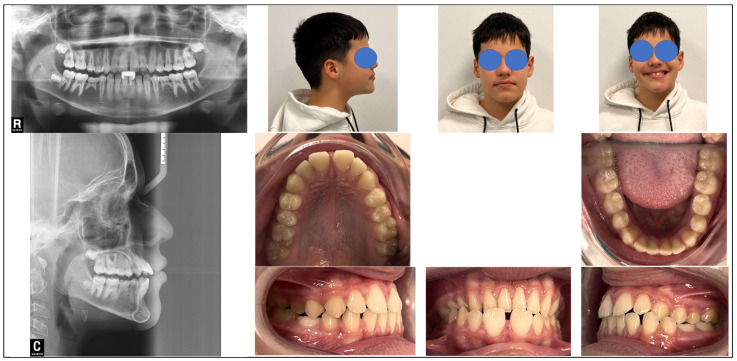
Clinical and radiographic examination. Upper left, panoramic radiograph. Lower left, cephalometric radiograph with Trevisi analysis. Upper right, extraoral pictures. Lower right, intraoral pictures.

Anamnesis, intraoral photographs and study dental casts were collected. Intraoral and extraoral examination was performed, Figure 2. Panoramic X-ray and lateral teleradiography was taken with Vatech PaX-400C (Hwaseong-si, Gyeonggi-do, Republic of Korea), and using the software EasyDent V4 Viewer version 4.1.5.10. Trevisi cephalometric analysis was conducted with the software Nemostudio version 22.0.1.220—UDI 8437023642078 (Nemotec, Madrid, Spain).

### 2.3. Data Analysis

The Flir tools software (Version 6.4.18039.1003) was used to obtain the temperature of the following regions of interest:

- Left and right temporal muscle;
- Left and right masseter muscle;
- Left and right upper orbicular oris muscle;
- Left and right lower orbicular oris muscle.

A datasheet was built in Microsoft Excel to obtain the temperature difference [ΔΤ] between left and right structures. Absolute values of ΔΤ greater than 0.3 °C in the ROI were considered “affected”, while absolute values of ΔΤ equal or inferior to 0.3 °C in the ROI were considered “normal”.

The Trevisi cephalometric parameters that were taken into consideration for the study were the following:

- SNA;
- SNB;
- ANB;
- OccltoSn;
- Wits relation to base;
- Molar and canine classes.

**Figure 3:**
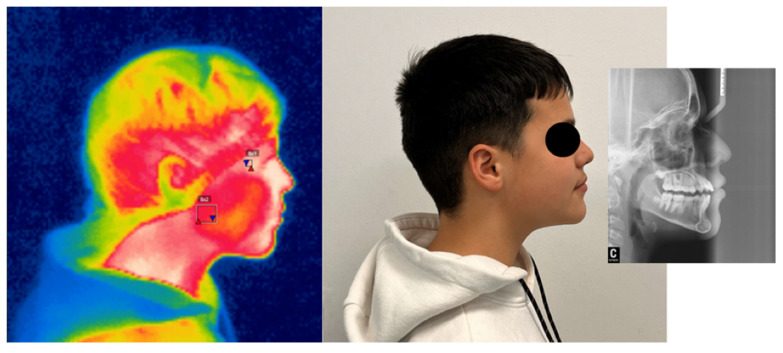
Combined method of imaging modality in orthodontics: infrared thermography and cephalometry.

Figure 3 shows the combination of infrared thermography, visible and cephalometry.

### 2.4. Statistical Analysis

IBM SPSS statistics version 27.0 was used to obtain data descriptive analysis (mean, standard deviation, maximum and minimum) and run statistical tests (Fisher exact tests) with an α of 5%. Fisher’s exact test was preferred since the total sample size was small (*n* = 22), expected frequency in any cell of 2 × 2 contingency table was less than 5. The data were nominal and their association or independence were tested.

## 3. Results

The data collected was computed and analyzed to obtain the following results. Firstly, for each ROI the minimum, maximum, mean and standard deviation (Table 1). Secondly, by comparing the temperature from the bilateral structures the ΔΤ was recorded. For the structures with a value over 0.3 °C was considered affected and for the rest considered normal (Table 2). Table 3, Table 4, Table 5 and Table 6 presents the frequencies of the studied cephalometric landmarks (SNA, SNB, ANB, OccltoSN, Wits, base relation, molar and canine relationship).

### Statistical Tests

Most of the participants showed a normal temperature difference ΔΤ for the upper and lower orbicular oris muscle, right vs. left, 96% and 92%, respectively. Thus, the following Fisher’s tests were performed for the masseter, temporal muscles and orbicular oris (Orb. oris superior vs. inferior), Table 7:

## 4. Discussion

IRT, as a medical imaging technology, can be applied to the oro-maxillo-mandibular region for the observation of certain regions such as the anterior temporalis muscle, the masseter muscle, the temporomandibular joint and the orbicularis muscle. The premise of this study is to analyze the thermal patterns, in particular the asymmetries to correlate with the patient’s clinical history and clinical examination. Namely, verifying whether these patterns actually correspond to alterations in the patient’s craniofacial morphology, or more specifically in the area of TMD, something that has been studied and described in the literature.

There have been enormous advancements in dentistry technology, clinical procedures and materials. Today oral health professionals have a wide range of options to use in the clinical environment to support the diagnosis and treatment of orthodontics, e.g. intraoral 3D scanners [13,14,15,16]. Orthodontics can be widely explored in terms of anatomical landmarks, biological data using metabolic fingerprint of saliva for bone growth and tooth development estimation [17] or physiological data regarding the muscle activity [18,19,20], or even implementing the use of infrared thermography to analyze and quantify anatomo-physiological reactions of specific areas correspondent to important structures of the cranio-cervico-mandibular complex which have a particular influence on dental and skeletal morphology [7,21,22]. Taking into consideration for example that most of the studies available report a skeletal asymmetric growth, and that EMG activity of masticatory muscles is different between crossbite and non-crossbite sides [20], it is interesting to use IRT to evaluating these issues, as the thermal patterns can allow clinicians to understand the asymmetry, for example, between the masseter muscle of a crossbite side and non-crossbite side. With our investigation, we intend to demonstrate what other authors also showed to be relevant, like in the research carried out by Nickel et al. [23], analyzing how the mechanic behavior of the temporomandibular joint loads and jaw muscle use was different between facial types and correlated with ramus height (Condylon–Gonion), being able to obtain results that showed significant differences between facial types when correlated with ramus height [23]. The characterization of muscle behavior is of common interest in orthodontics, where Deguchi et al. demonstrated that, when comparing with normal subjects, patients with a Class III malocclusion have a demonstrable abnormal masticatory muscle balance which is well characterized by EMG [24]. Infrared thermography can thereby be an interesting tool to determine such asymmetries enhancing the thermal pattern of the neuromuscular activity in the correspondent zone of the muscle (without the need of electrode placement). Tosello et al. [25] analyzed the EMG activity of orbicularis oris upper and lower, and the mentalis muscles during several movements of the lips, in 18 children ageing from 8 to 12 years, divided into three groups: one with normal occlusion, and two with class II division 1, with atypical swallowing and/or incompetent lips who had received no orthodontic treatment, showing asymmetrical patterns of muscle activity [25]. The comprehension of these issues is crucial for an orthodontic treatment and its stability post-orthodontic. For example, when evaluating lip incompetence, Dei et al.’s findings suggest that observing blood flow in the mentalis muscle is an effective and easily performed method using laser speckle imaging [26]. Apart from lip incompetence, the possible interaction of breathing disorders in the etiology of malocclusion is also widely known, and Zhao Minyue et al. highlighted that breathing modes and vertical skeletal patterns interacted with alter maxillofacial EMG activities [26].

Masticatory function, including muscle activity and occlusal function, can be affected by craniofacial morphology [27]. Yoon Yu concluded that vertical craniofacial morphology was related to masticatory function. Hypodivergent individuals may have low masticatory muscle activity and high occlusal function, resulting in good masticatory muscle efficiency [27]. Alabdullah et al. [28] found a significant relationship between facial muscle activity and facial growth pattern, showing that their findings suggest the activity of masticatory and perioral muscles could play a role in the facial growth. The implementation of a complementary diagnostic system, with thermal imaging, as an addition to Angle’s classification, cephalometry, intra-oral, extra-oral photographs and dental casts analyses not only defines the natures but can also contribute to identifying the morpho-functional developmental mechanisms of malocclusions.

Nowadays, artificial intelligence (AI) is starting to fill in the gap in orthodontic–orthognathic treatment, helping the clinician in the planning of complex cases [29]. Explainable AI components can make complex model decisions interpretable within clinical contexts using an interactive collaborative human–AI [29]. The multifactorial and multivariable nature of malocclusion makes any single classification not only difficult but also of limited value in dentofacial assessment [30]. On a daily basis, orthodontics has improved its diagnostic field using CBCT over conventional radiography, thus facilitating the appropriate treatment planning of patients [31].

Apart from all these considerations, it is also important to collect the psychological signs of a patient that will be submitted to an orthodontic treatment [32], so analyzing the psychosomatic profile of an individual should also be a matter of interest in orthodontics. Besides this, temporomandibular disorders should be a part of an exhaustive clinical history and examination of the patient being submitted to an orthodontic treatment [29]. In the area of TMD, infrared thermography can be an important diagnostic tool.

The use of thermography as a complementary diagnostic method in the identification of TMD can be effective [7,33], but some authors point out that there is still a lack of evidence in the literature in terms of studies that defend the effectiveness of this technique in the diagnosis of TMD. It is therefore necessary to understand some of the technique’s limitations as well as its advantages. In the case of patients with parafunctional masticatory habits, such as bruxism, which causes hyperactivity of the masseter and temporalis muscles and can lead to imbalances in the blood circulation of the muscles in question, with the activation and sensitization of nociceptors in the central nervous system, causing muscle pain. IRT is an imaging method that can identify a pattern associated with pain in the oromaxillomandibular region. However, we can characterize and visualize possible alterations in structures that are important to us as clinicians, researchers and teachers. Thermography is a medical imaging modality that can be introduced into the daily routine of a health professional dedicated in particular to the study and treatment of craniofacial dysmorphia, more specifically in an orthodontic consultation, as in this study, carried out in the Lisbon Pediatric Dentistry Service [SOL] of the Santa Casa da Misericórdia de Lisboa.

Orthopantomography is certainly the most commonly used diagnostic aid in dentistry; it makes possible to visualize tooth parts, the bony structures of the maxilla and mandible, any beginnings of condyle flattening, changes in the inclination of the articular eminence, asymmetries of the mandibular branches, the relationship of the maxillary sinuses with the tooth roots or even with the bone tissue in the case of edentulous areas, the proximity of the inferior alveolar nerve to the teeth (third molar included) which can sometimes be closely related, the detection of intraosseous lesions, among others. In this study, the authors wanted to introduce the infrared imaging technique in an orthodontic consultation, as a complementary diagnostic aid, in addition to the standard protocol established for craniofacial dysmorphia cases, namely in terms of complementary diagnostic means, lateral teleradiographs, computed tomography, intra-oral and extraoral photographs and study models. In this context, the use of thermography seems to be an asset as it is a non-invasive test and does not emit radiation since it captures the infrared radiation emitted by the human body. This infrared radiation is detected by the thermographic camera, making it possible to observe the anatomical–physiological reactions that are present in the musculoskeletal system in the face of existing changes in the vascular system, thus correlating the different skeletal patterns and interrelationships of the craniofacial morphology of the patient presenting for an orthodontic consultation.

The present sample showed a balanced temperature between left and right ROIs (Table 1), although there were individuals showing asymmetric ROIs (Table 2), with temperature differences at the level of hundredths, which is characteristic of a population without muscular temporomandibular disorders. Benedikt et al. enabled the application of modern landmark-based morphometrics to characterize biologic and craniofacial development. They discussed the theoretical foundations of morphometrics as applied to development and reviewed the basic approaches to the quantification of morphology, when introducing volumetric imaging for this analysis [34]. Of course, the basis of cephalometry has been widely discussed since the development of X-ray technology and its application to the craniofacial skeleton, and numerous landmarks have been described and studied to better diagnose and plan treatment options for anomalies related to facial architecture [35]. Retrognathic facial type, anterior dentoalveolar height, maxillary body length, cranial base and facial width, mandibular ramus height, anterior maxillary body height, mandibular length, cranial vault height, vertical position of the condyles which have been widely analyzed within the craniofacial complex [36]. Manolis et al. compared the craniofacial morphology in ancient and modern Greeks through 4000 years analyzing skulls and their respective cephalograms in order to determine geometric morphometrics of this population indicating elements of ethnic group continuation within the inevitable multicultural combinations [36]. So apart from different assessing imaging techniques, throughout time, embracing different populations, craniofacial morphology is a matter of interest in modern dentistry where all information collected by the clinician can be valuable in terms of treatment plan. Therefore, in the present study the authors applied infrared thermography, analyzing and crossing data of different cephalometric landmarks. The null hypothesis was accepted since no statistically significant differences were verified. None of the ROIs studied showed an asymmetrical pattern with statistical significancy when comparing different cephalometric landmarks of craniofacial morphology. The authors would like to highlight that, in the present results, there was a tendency for the infrared findings regarding the ROI of masseter muscle to be related to the OccltoSN parameter (*p* = 0.070) and the ROI of orbicular oris muscle (superior vs. inferior) to be related to SNB (*p* = 0.056). Regarding these results, the OccltoSN is a parameter associated to the vertical skeletal and dental relationships, obtained with the angle between the occlusal plane (the plane of contact between the upper and lower teeth) and the Sella-Nasion (SN) plane, a reference line in the skull. This cephalometric pattern is related to a clockwise hyperdivergence pattern that in orthodontics refers to a specific facial growth pattern characterized by a downward and backward rotation of the mandible (lower jaw) relative to the skull base, resulting in a longer lower face height and a more vertical facial appearance. In contrast, the hypodivergence pattern is a counterclockwise rotation, which involves an upward and forward rotation. These features support the notion that craniofacial morphology is associated with differences in the neuromuscular activity of the masticatory muscles. Thus, in the authors’ opinion, the implementation of infrared thermography can be a useful tool in a complementary diagnosis of structures that present, for example, relationships among interocclusal distances, mandibular plane angles and facial height. Raadsheer et al. showed that bite force magnitude was correlated significantly positively with vertical and transverse facial dimensions and the inclination of the midface, and significantly negatively with mandibular inclination and occlusal plane inclination, where the contribution of the masseter muscle to the variation in bite force magnitude was higher than that of the craniofacial factors [37]. So, independently of having the ultrasonography acquisition of the thickness of the masseter muscle having been performed by Raadsheer, when analyzing the contribution of jaw muscle size to the craniofacial morphology, it can be relevant to analyze the anatomical–physiological perspective within the same areas of interest with infrared thermography. This technique with an infrared camera is complementary to other techniques like electromyographic recording that have been widely applied when investigating relationships in craniofacial morphology and masticatory muscle activity [38].

When studying a malocclusion, it is also relevant to understand the biomechanics of the temporomandibular joint and the consequent influence of craniofacial morphology on mandibular border movements. Kataoka et al. assessed one hundred female subjects which were selected from outpatients visiting the orthodontic clinic of Okayama University Hospital. Their study showed that craniofacial morphology had different influences on each mandibular border movement. In particular, during maximum jaw laterotrusion, lower incisor movement strongly reflected condylar movement, and the influence of craniofacial morphology on mandibular border movement was minimal [39]. Taking this into consideration it is worthwhile to consider the use of infrared thermography in the study of malocclusion, independently of the results of our investigation which has not been able to show significantly statistical relevant results, probably due to a reduced sample. The authors believe that, in severe craniofacial dysmorphias, infrared thermography could correlate with specific cephalometric landmarks being an added value in the area of diagnosis at an orthodontic center. Considering the results of the thermograms for the orbicular oris muscles, the asymmetric values were more prevalent in patients with a mandibular retrusion (7 individuals out of 22) when correlating with the SNB parameter. It seems that the thermal behavior of the superior orbicular oris muscle might be different from the inferior orbicular oris muscles when comparing individuals with different mandible positions (prognatic, normal or retrognatic). On the other hand, when comparing the thermal values between the left and right regions of each respective orbicular oris (superior and inferior), the number of asymmetries is reduced. Future studies in this specific area should favor their research on a broader sample, and compare the thermograms of temporal, masseter and orbicularis oris muscles for retrognatic/prognatic patients and hyper/hypodivergent facial patterns.

This investigation sheds light on a new approach to orthodontic treatment planning, as it allows for a noninvasive diagnostic examination of certain regions of interest in oromaxilomandibular muscles. The relevance of electromyographic devices is well known, for example, in assessing the activity of the orbicular muscle in interceptive orthodontics, where improvements in muscle contraction after treatment have been demonstrated by surface electromyography. This electromyographic technique was also employed by Suzane et al., suggesting the existence of different profiles of muscle activation in the orbicularis oris muscle during the suction of various liquids in breastfed and non-breastfed children [40]. It is extremely relevant for a pediatric center like SOL, part of the Santa Casa da Misericórdia de Lisboa, which attends ten thousand patients per year, to correlate clinical findings with exhaustive anamneses where functional habits, such as atypical swallowing, can be correlated with the etiological factors of developing a malocclusion. For this purpose, quantifying thermal patterns of the orbicularis oris muscles should be an important step toward quantifying the existing physiological and anatomical features regarding these structures that have direct implications in dental, skeletal and craniofacial morphology. Bregnoni et al. investigated the influence of atypical swallowing on skeletal and alveolar development by performing 100 cephalometric analyses on a group of patients with this alteration in swallowing and a control group of another 100 patients without atypical swallowing. It was possible to conclude from their study that atypical swallowing affected skeletal growth, causing mandibular clockwise rotation, skeletal Class II, open bite and incisor proclination. Begnoni et al. also verified that, to compensate for these effects, an increase in alveolar growth, together with molar eruption, seems to be induced [41]. The findings of our current investigation can highlight asymmetric patterns of the orbicularis oris muscle when comparing the lower and upper orbicularis oris, which has indeed been a matter of interest along with the masticatory muscles when we address the subject of orthodontic treatment in Class II Division 1 patients. Tancan et al. evaluated the effects of the pre-orthodontic trainer (POT) appliance on the anterior temporal, masseter, mental and orbicularis oris muscles through electromyographic evaluations in patients with Class II Division 1 malocclusion and incompetent lips [42]. Electromyography records were taken during different oral functions such as clenching, sucking and swallowing, making it possible to observe that, in the group treated with the pre-orthodontic trainer treatment, the activity of the anterior temporal, mental and masseter muscles decreased while the orbicularis oris activity increased during clenching. In their study, these differences were found to be statistically significant when compared to the control group. The orbicularis oris activity during sucking was also increased in the treatment group (*p* < 0.05). Other authors, namely Ahmet Yagci et al., also analyzed electromyographic recordings of the masticatory and perioral muscles of Class II Division 1 patients before and after a myofunctional appliance, observing a decrease in electromyographic values for the orbicularis oris muscle during sucking (*p* < 0.05) and clenching (*p* < 0.01) [43].

Taking this into account, clinicians and other researchers have been paying attention to the orbicularis oris, something that our current investigation intends to advocate with the introduction of infrared thermography and its importance as an imaging technique at the beginning of orthodontic treatment since it can ensure any eventual asymmetric pattern of the temperature differential between the upper and lower orbicularis oris. This will not only allow the clinician to interpret any eventual necessity for re-education of the lower lip regarding parafunctional activity but also identify the presence of hyperfunction of this structure, which could be seen as a higher temperature value when compared to the upper lip. A particular focus on these features will also enable the orthodontist to ensure better stability post-orthodontic treatment since the activity and eventual pressures/forces of the perioral structures can be identified with the aid of thermal patterns. Even in cases of frenectomy of the tongue, rehabilitation exercises seem to affect the function of the orofacial muscles, as shown by a study conducted by Simona et al., using electromyography [44]. Interestingly, it is noteworthy that the upper and lower fascicles of the orbicularis oris muscle in edentulous patients were compared before and after complete implantation, where the significance of these structures in the constitution of a morpho-functional system of the lower third of the face impacts the clinical determinations of the occlusal vertical dimension. Moreto Santos et al. showed, for the muscles factor, higher electromyographic readings in the lower fascicle of the orbicular oris muscle, as compared with those of the upper fascicle. The comparison among the clinical conditions indicated higher electromyographic values for the edentulous condition (i.e., before complete denture implantation), as compared to those recorded after denture implantation [45]. In another study in the area of oral rehabilitation, Moreira et al. applied infrared thermography to assess the thermal effect of prosthodontic treatment on the structures of the cranio-cervico-mandibular complex, where it was possible to conclude that the prosthodontic treatment has a minimal or negligible effect on the temperature differential of the ROIs of the temporomandibular joint and the temporal muscles. On the other hand, the orbicularis muscles exhibited significant thermal variations [46].

The data interpreted in this study show that, nowadays, the use of infrared thermography can complement the cephalometric analysis that assesses the skeletal or dentoalveolar basis of malocclusion. The interpretation of anatomo-physiological features regarding the perioral structures, like the orbicularis oris, will enable the orthodontist to enhance the diagnosis of the patient concerning the soft tissues and specific ROIs. Even with the increased use of imaging techniques like CBCT equipment to develop cephalometric analyses, improving the diagnostic value from 2D to 3D cephalometric analyses, there are anatomical regions that cannot be measured, observed or quantified. This can be overcome with the implementation of infrared thermography in the analysis of craniofacial morphology during an orthodontic consultation.

## 5. Conclusions

In this sample, no statistically significant associations were found between the thermal state of the temporalis, masseter and orbicularis oris muscles and Trevisi cephalometric landmarks (SNA, SNB, ANB, OccltoSN, Wits, base relation, molar and canine relationship).

It is relevant to consider the importance of parafunctional activities, such as atypical swallowing, in which the force exerted by the lower orbicularis oris may result in higher temperature values compared with the upper orbicularis oris. The significance of these findings lies in the fact that asymmetric patterns detected by infrared thermography can serve as an aid in diagnosis and orthodontic treatment planning, since the long-term stability of post-orthodontic treatment is highly dependent on the muscular activity of the tongue and lips—particularly in patients with atypical swallowing.

The analysis of the cranio-cervico-mandibular complex and other features quantified by infrared thermography, such as posterior crossbite and dentoskeletal relationships, may in the future complement the diagnostic tools that orthodontists already use in daily practice.

Further studies with larger patient samples are needed to confirm the usefulness of thermal imaging in orthodontic populations.

Our findings suggest that this technique can be used to quantify anatomical landmarks relevant to craniofacial morphology in specific populations, particularly at ages where muscular functional activity is strongly correlated with dentoskeletal development.

## Figures and Tables

**Table 1:** Descriptive analysis of temperature of the studied ROIs.

ROI	N	Minimum	Maximum	Mean	Std. Deviation
Left Temporal	18	31.50	34.20	33.53	0.648
Right Temporal	18	32.70	34.90	33.57	0.549
Left Masseter	22	30.90	34.20	32.54	0.911
Right Masseter	22	31.00	34.00	32.51	0.797
Right upper O. oris	22	32.20	34,70	33.53	0.677
Left upper O. oris	22	32.32	35.00	33.53	0.657
Left lower O. oris	22	32.30	34.70	33.46	0.575
Right lower O. oris	22	31.90	34.70	33.52	0.654

**Table 2:** Frequency tables for the thermographic pattern [normal/affected] of each ROI.

	Frequency	Percentage (%)
**Temporal Muscle**		
Normal	10	45.5
Affected	9	36.4
Total	18	81.8
Missing	4	18.2
**Masseter muscle**		
Normal	10	45.5
Affected	12	54.5
Total	22	100
**Upper orbicular oris muscle [Right vs. Left]**		
Normal	21	95.5
Affected	1	4.5
Total	22	100
**Inferior orbicular oris muscle [Right vs. Left]**		
Normal	20	90.9
Affected	2	9.1
Total	22	100
**Orbicular oris muscle [Superior vs. Inferior]**		
Normal	15	68.2
Affected	7	31.8
Total	22	100

Note: ROI was considered affected when the level of asymmetry was superior to 0.30 °C.

**Table 3:** Frequency tables for the studied cephalometric landmarks (SNA and SNB).

	SNA		SNB	
	Frequency	%	Frequency	%
Retrognatic	11	50.0	13	59.1
Norma	6	27.3	6	27.3
Prognatic	5	22.7	3	13.6
Total	22	100	22	100

**Table 4:** Frequency tables for the studied cephalometric landmarks (ANB and Wits).

	ANB		Wits Relation to Base	
	Frequency	%	Frequency	%
Class I	7	31.8	7	31.8
Class II	8	36.4	6	27.3
Class III	7	31.8	9	40.9
Total	22	100	22	100

**Table 5:** Frequency tables for the studied cephalometric landmarks (OccltoSN).

	OccltoSN	
	Frequency	%
Clockwise	17	77.3
Normal	2	9.1
Counterclockwise	3	13.6
Total	22	100

**Table 6:** Frequency tables for the studied cephalometric landmarks (Left / Right Canine and Molar).

	Left Canine Relationship		Right Canine Relationship		Left Molar Relationship		Right Molar Relationship	
	Frequency	%	Frequency	%	Frequency	%	Frequency	%
I	14	63.6	12	54.5	13	59.1	12	54.5
II	5	22.7	7	31.8	6	27.3	7	31.8
III	1	4.5	1	4.5	1	4.5	1	4.5
Total	20	90.9	20	20.9	20	90.9	20	90.9
Missing	2	9.1	2	9.1	2	9.1	2	9.1

**Table 7:** Statistical tests.

	SNA	SNB	ANB	OccltoSN	Wits Relation to Base	Right Molar Relationship	Left Molar Relationship	Right Canine Relationship	Left Canine Relationship
Masseter	0.296	0.340	0.867	0.070	0.486	1.000	0.336	0.259	0.796
Temporal	0.148	0.397	0.292	1.000	0.827	1.000	0.424	1.000	1.000
Orb. oris [sup.inf]	1.000	0.167	0.614	0.565	0.845	1.000	1.000	0.777	0.742

No statistically significant differences were found.

## Data Availability

The data presented in this study are available upon request from the corresponding author due to privacy and ethical reasons.

## Abbreviations

IRT (Infrared Thermography), ROIs (Regions of interest), SOL (Lisbon Pediatric Dentistry Service), TMDs (temporomandibular disorders).

## References

[1] Merla A., Romani G.L. Functional Infrared Imaging in Medicine: A Quantitative Diagnostic Approach. In Proceedings of 2006 International Conference of the IEEE Engineering in Medicine and Biology Society.

[2] Schlager O., Gschwandtner M.E., Herberg K., Frohner T., Schillinger M., Koppensteiner R., Mlekusch W. (2010). Correlation of infrared thermography and skin perfusion in Raynaud patients and in healthy controls. Microvasc. Res..

[3] Liu C., van Netten J.J., van Baal J.G., Bus S.A., van der Heijden F. (2015). Automatic detection of diabetic foot complications with infrared thermography by asymmetric analysis. J. Biomed. Opt..

[4] Jones B.F. (1998). A reappraisal of the use of infrared thermal image analysis in medicine. IEEE Trans. Med. Imaging.

[5] Ring E.F., Ammer K. (2012). Infrared thermal imaging in medicine. Physiol. Meas..

[6] Nahm F.S. (2013). Infrared thermography in pain medicine. Korean J. Pain..

[7] Pais Clemente M., Azevedo Coutinho F., Mendes J., Pinto J., Amarante J.M. The Application of Infrared Thermography as a Quantitative Sensory Measure of the DC/TMD. Proceedings of the ECCOMAS Thematic Conference on Computational Vision and Medical Image Processing.

[8] Moreira A., Batista R., Oliveira S., Branco C.A., Mendes J., Figueiral M.H. (2021). Role of thermography in the assessment of temporomandibular disorders and other musculoskeletal conditions: A systematic review. Proc. Inst. Mech. Eng. H.

[9] Ammer K. (2008). The Glamorgan Protocol for recording and evaluation of thermal images of the human body. Thermol. Int..

[10] Howell K., Re S. (2009). Guidelines for specifying and testing a thermal camera for medical applications. Thermol. Int..

[11] Le T., Tran P., Tran V. (2022). Factors that affect lip changes following incisor retraction in Vietnamese adults with a convex facial profile. J. Orthod. Sci..

[12] Punyanirun K., Charoemratrote C. (2025). Lower lip changes after overjet reduction with and without mandibular incisor retraction. Am. J. Orthod. Dentofac. Orthop..

[13] Cen Y., Huang X., Liu J., Qin Y., Wu X., Ye S., Du S., Liao W. (2023). Application of three-dimensional reconstruction technology in dentistry: A narrative review. BMC Oral Health.

[14] Jheon A.H., Oberoi S., Solem R.C., Kapila S. (2017). Moving towards precision orthodontics: An evolving paradigm shift in the planning and delivery of customized orthodontic therapy. Orthod. Craniofacial Res..

[15] Frèrejouand E. (2016). 3D imaging benefits in clinical pratice of orthodontics. Orthod. Fr..

[16] Kapila S., Conley R.S., Harrell W.E. (2011). The current status of cone beam computed tomography imaging in orthodontics. Dentomaxillofacial Radiol..

[17] Tsagkari E., Deda O., Krokos A., Gika H., Papadopoulos M.A., Chatzigianni A. (2022). Investigation of salivary biomarkers as indicators of skeletal and dental maturity in children. Orthod. Craniofacial Res..

[18] Woźniak K., Piątkowska D., Lipski M., Mehr K. (2013). Surface electromyography in orthodontics—A literature review. Med. Sci. Monit..

[19] Szyszka-Sommerfeld L., Lipski M., Woźniak K. (2020). Surface Electromyography as a Method for Diagnosing Muscle Function in Patients with Congenital Maxillofacial Abnormalities. J. Healthc. Eng..

[20] Iodice G., Danzi G., Cimino R., Paduano S., Michelotti A. (2016). Association between posterior crossbite, skeletal, and muscle asymmetry: A systematic review. Eur. J. Orthod..

[21] Haddad D.S., Brioschi M.L., Baladi M.G., Arita E.S. (2016). A new evaluation of heat distribution on facial skin surface by infrared thermography. Dentomaxillofacial Radiol..

[22] Clemente M.P., Mendes J., Vardasca R., Moreira A., Branco C.A., Ferreira A.P., Amarante J.M. (2020). Infrared thermography of the crânio-cervico-mandibular complex in wind and string instrumentalists. Int. Arch. Occup. Environ. Health.

[23] Nickel J.C., Weber A.L., Covington Riddle P., Liu Y., Liu H., Iwasaki L.R. (2017). Mechanobehaviour in dolichofacial and brachyfacial adolescents. Orthod. Craniofacial Res..

[24] Deguchi T., Garetto L.P., Sato Y., Potter R.H., Roberts W.E. (1995). Statistical analysis of differential lissajous EMG from normal occlusion and Class III malocclusion. Angle Orthod..

[25] Tosello D.O., Vitti M., Berzin F. (1999). EMG activity of the orbicularis oris and mentalis muscles in children with malocclusion, incompetent lips and atypical swallowing—Part II. J. Oral. Rehabil..

[26] Dei A., Miyamoto J.J., Takada J., Ono T., Moriyama K. (2016). Evaluation of blood flow and electromyographic activity in the perioral muscles. Eur. J. Orthod..

[27] Yoon Y.J., Kang J.Y., Kim K.H., Cha J.Y., Ahn H.J., Choi Y.J. (2023). Correlation of masticatory muscle activity and occlusal function with craniofacial morphology: A prospective cohort study. Clin. Oral. Investig..

[28] Alabdullah M., Saltaji H., Abou-Hamed H., Youssef M. (2015). Association between facial growth pattern and facial muscle activity: A prospective cross-sectional study. Int. Orthod..

[29] Ehrmann E., Bernabeu M., Charavet C. (2024). Temporomandibular Disorders and Orthodontics: How to conduct a screening examination?. Orthod. Fr..

[30] Otuyemi O.D., Jones S.P. (1995). Methods of assessing and grading malocclusion: A review. Aust. Orthod. J..

[31] Merrett S.J., Drage N.A., Durning P. (2009). Cone beam computed tomography: A useful tool in orthodontic diagnosis and treatment planning. J. Orthod..

[32] Benkimoun F. (2022). Collecting psychological signs in the diagnosis of skeletal dysmorphoses. Orthod. Fr..

[33] Woźniak K., Szyszka-Sommerfeld L., Trybek G., Piątkowska D. (2015). Assessment of the Sensitivity, Specificity, and Accuracy of Thermography in Identifying Patients with TMD. Med. Sci. Monit..

[34] Hallgrimsson B., Percival C.J., Green R., Young N.M., Mio W., Marcucio R., Chai Y. (2015). Chapter Twenty—Morphometrics, 3D Imaging, and Craniofacial Development. Current Topics in Developmental Biology.

[35] Taub P.J. (2007). Cephalometry. J. Craniofacial Surg..

[36] Liebgott B. (1977). Factors of human skeletal craniofacial morphology. Angle Orthod..

[37] Raadsheer M.C., van Eijden T.M.G.J., van Ginkel F.C., Prahl-Andersen B. (1999). Contribution of Jaw Muscle Size and Craniofacial Morphology to Human Bite Force Magnitude. J. Dent. Res..

[38] Takeuchi-Sato T., Arima T., Mew M., Svensson P. (2019). Relationships between craniofacial morphology and masticatory muscle activity during isometric contraction at different interocclusal distances. Arch. Oral Biol..

[39] Kataoka T., Kawanabe N., Shiraga N., Hashimoto T., Deguchi T., Miyawaki S., Takano-Yamamoto T., Yamashiro T. (2013). The Influence of Craniofacial Morphology on Mandibular Border Movements. CRANIO®.

[40] Jacinto-Gonçalves S.R., Gavião M.B., Berzin F., de Oliveira A.S., Semeguini T.A. (2004). Electromyographic activity of perioral muscle in breastfed and non-breastfed children. J. Clin. Pediatr. Dent..

[41] Begnoni G., Cadenas de Llano-Pérula M., Dellavia C., Willems G. (2020). Cephalometric traits in children and adolescents with and without atypical swallowing: A retrospective study. Eur. J. Paediatr. Dent..

[42] Uysal T., Yagci A., Kara S., Okkesim S. (2012). Influence of pre-orthodontic trainer treatment on the perioral and masticatory muscles in patients with Class II division 1 malocclusion. Eur. J. Orthod..

[43] Yagci A., Uysal T., Kara S., Okkesim S. (2010). The effects of myofunctional appliance treatment on the perioral and masticatory muscles in Class II, Division 1 patients. World J. Orthod..

[44] Tecco S., Baldini A., Mummolo S., Marchetti E., Giuca M.R., Marzo G., Gherlone E.F. (2015). Frenulectomy of the tongue and the influence of rehabilitation exercises on the sEMG activity of masticatory muscles. J. Electromyogr. Kinesiol..

[45] Santos C.M., Vitti M., de Mattos Mda G., Semprini M., Paranhos Hde F., Regalo S.C. (2003). Electromyographic analysis of the upper and lower fascicles of the orbicular oris muscle, in edentulous patients, before and after complete denture implantation. Electromyogr. Clin. Neurophysiol..

[46] Moreira A., Batista R., Oliveira S., Mendes J., Sampaio-Fernandes M., Figueiral M.H. (2021). The Thermal Influence of Oral Rehabilitation on the Cranio-Cervico-Mandibular Complex: A Thermographic Analysis. Int. J. Environ. Res. Public Health.

